# Phases of Match-Play in Professional Australian Football: Positional Demands and Match-Related Fatigue

**DOI:** 10.3390/s22249887

**Published:** 2022-12-15

**Authors:** Michael J. Rennie, Stephen J. Kelly, Stephen Bush, Robert W. Spurrs, William B. Sheehan, Mark L. Watsford

**Affiliations:** 1 School of Sport, Exercise and Rehabilitation, Faculty of Health, University of Technology Sydney, Moore Park, Sydney, NSW 2021, Australia; 2 Sydney Swans Football Club, Moore Park, Sydney, NSW 2021, Australia; 3 School of Mathematics and Physical Sciences, Faculty of Science, University of Technology Sydney, Ultimo, NSW 2007, Australia

**Keywords:** physical performance, time-motion analysis, activity profiles, position, GPS, GNSS

## Abstract

This study examined the influence of player position and match quarter on activity profiles during the phases of play in Australian Football. Global positioning satellite data was collected for one season from an Australian Football League team for nomadic, key position and ruck players (age: 24.8 ± 4.2 years, body mass: 88.3 ± 8.7 kg, height: 1.88 ± 0.8 m). Separate linear mixed models and effect sizes were used to analyse differences between positions and game quarter within each phase of play for values of distance, speed and metabolic power indices. There were clear differences between positions for low-speed running, high-speed running, total distance and average speed. Nomadic players generally recorded the highest match running outputs, followed by key position players and ruckmen. Within each position, offence and defence involved the highest intensities, followed by contested play and then stoppage periods. Across the four quarters, there were small to large reductions in average speed, high-speed running, high power and energy expenditure during offence, defence and contested play, but not during stoppages. Accordingly, conditioning staff should consider the intermittent intensities of the phases of match-play for each position to optimally prepare players for competition. Reductions in match intensities were evident during active periods of play providing implications for real-time monitoring to optimise the timing of rotations.

## 1. Introduction

Time motion analysis research in professional Australian Football (AF) has evolved progressively over the last 40 years and has been integral in understanding the high intensity, intermittent nature of competitive match-play [1]. The breadth of analysis has increased with advancements in technology, whereby now all teams in the Australian Football League (AFL) utilize global positioning satellite (GPS) technology to quantify the physical performance of their players. Depending on position, players typically cover total distances of 11–14 km, with 22–33% performed above 14.4 km·h^−1^ [2]. Furthermore, there is a consensus in the literature that match-running performance is influenced by a myriad of dynamic factors including match-related fatigue [3], playing position [4], player caliber [5], quarter outcome [6] and technical proficiency [7,8,9].

Recently, the integration of video analysis and GPS has elicited the ability to assess specific elements of match-play that may provide more robust information compared to entire match or quarter-by-quarter analyses. Several studies in English Premier League (EPL) and Spanish La Liga have shown that match-running profiles are influenced by both playing position and phase of match-play [10,11,12]. Typically, these studies have assessed periods of offence and defence and reported disparate findings. For example, central attacking midfielders in the La Liga performed more high-speed running (HSR) during offence, however the opposite was found for central attacking midfielders in the EPL [11]. In contrast, fixed position players (central forwards and defenders) typically performed the majority of running that was reflective of their positional role (i.e., forwards performed more HSR during offence) [11]. Research from professional rugby league has demonstrated that while defending was found to be more physically demanding overall when compared to offence [13], this was only the case for forwards and pivots with no difference for outside backs [14]. This information indicates that match running profiles may be dependent on the players’ position and specific tactical role within the team.

Early time motion analysis studies in team sports primarily used manual notation methods by individually tracking player movement using a video recording [15]. These methods were highly laborious and therefore quantifying the movement profiles of large sample sizes were challenging. Movement analysis has now progressed to automated video motion systems that are non-invasive and can be used to analyse movement, technical skills and tactical information [10,16]. The majority of contemporary research in AF now adopts the use of GPS which can provide speed and distance-based indices, accelerometry and metabolic power information. Indeed, several AF studies have described the influence of player position on the physical demands of match-play using GPS technology [2,4,17]. Traditional time motion variables along with metabolic power estimates have shown that nomadic players typically perform the highest match-running outputs, followed by fixed defenders and forwards, and then ruck players [2,4]. While team physical characteristics have previously been assessed in different phases of match play [18,19], it has been acknowledged that each position has a specific role during different periods of the game, and it is therefore plausible that in-game demands may vary depending on the phase of play for each position. Knowledge of this information would be useful for designing specific training for the various positions in AF.

One area that has received detailed attention is the incidence of match-induced fatigue during AF match-play, which has typically been demonstrated by decrements in HSR and average speed. Several studies have utilised fixed time periods to assess transient and cumulative fatigue including: comparing the peak 3 min period of activity to the subsequent 3 min period and mean of all other 3 min periods [20], first player rotation period to subsequent rotation periods [21], and more recently, a moving average using ten different durations (1–10 min) with the maximum value recorded for each duration [22]. Collectively, these studies have demonstrated evidence of match-related fatigue following the most intense periods of match-play and during the final quarter of a game. Recently, authors have expressed caution when interpreting findings related to match-induced fatigue that do not account for time spent in possession or when the ball is out of play [16,23]. Theoretically, reductions in movement intensities may be the result of stoppages in match-play that primarily involve low-intensity activity [18,19]. In AF, high-intensity periods of offence, defence or contested play can be proceeded by stoppages of varying durations. These stoppages can be due to the ball travelling over the sideline or ‘held-up’ in the field of play, during a set shot for goal or a goal reset. Clearly, further knowledge can be fostered by analysing the phases of match-play during AF competition. Specifically, this analysis could identify (a) how the positional demands differ depending on the phase of match-play and (b) whether reductions in match-running performance are global or isolated to specific phases. Thus, the aim of this study was to assess the effect of the phases of AF match-play activity profiles relevant to player position and match quarter.

## 2. Materials and Methods

Thirty-three professional AFL players from one club participated in the study (age: 24.8 ± 4.2 years, body mass: 88.3 ± 8.7 kg, height: 1.88 ± 0.8 m, total match observations: 9.6 ± 5.3; mean ± SD). Eighteen in-season matches were analysed, eliciting 356 player files following exclusion criteria being applied. Players were categorized as either nomadic (n = 22 (227)), key position (n = 7 (85)) or ruck (n = 4 (44)) depending on their primary role for the match. University ethics approval, consent from the AFL club and participating players was obtained prior to data collection.

Movement data was collected using GPS technology (Optimeye S5, 10-Hz, Catapult Sports, Melbourne, Victoria, Australia) fitted into a custom-designed pouch at the back of the players’ jersey. Players wore the same GPS unit for each game to minimise inter-unit error. Time between quarter breaks and interchange periods was omitted prior to analysis. Players who played less than 75% of total match time were excluded from the analysis. For the positional analysis, data was categorised into total (m, TD) and average speed (m·min^−1^), while high-speed running (>14.4 km·h^−1^, HSR) and low-speed running (<14.4 km·h^−1^, LSR) were reported relative to field time (m·min^−1^). The validity and reliability of these GPS units and metrics have been previously reported [24,25]. The available satellites and horizontal dilution of precision (HDOP) were 18.2 ± 1.1 (range 14–19) and 1.1 ± 0.1 arbitrary units (AU) (range 0.5–1.2), respectively. Additionally, estimates of instantaneous metabolic power produced above a pre-determined high-power threshold (>20 W·kg^−1^, HP) and energy expenditure (kj·kg^−1^) were calculated using methods developed by others [26,27] and reported for phases of match-play and quarter for nomadic players.

Video footage was coded by a single researcher for phases of offence, defence, contested play, umpire stoppages, set shots and goal resets using SportsCode (SportsTec Limited, version 9.4.1, Warriewood, Australia). Intra-class correlation coefficients ranged from 0.992–0.865 for absolute reliability and intra-coder typical error of measurement was 6.9%, 8.2%, 9.3%, 9.0%, 2.7% and 1.8% for offence, defence, contested play, umpire stoppage, set shot and goal reset, respectively [28]. Following coding, a time-stamped transcript of the phases of play was uploaded to a Microsoft Excel spreadsheet (Microsoft, Redmond, WA, USA). The spreadsheet was imported into the GPS proprietary software and produced a total of 33,398, 33,642, 67,148, 35,204, 8252 and 7396 individual player transcripts for offence, defence, contested play, umpire stoppage, set shot and goal reset, respectively.

Data was analysed using a generalized linear mixed effects model (*lme4* package) to determine the interaction between phase of play, position and quarter for each physical performance metric. Each individual player was included as a random effect to account for pseudo-replication. The assumptions of normality were verified a priori for parametric analysis. Separate two-way analysis of variance (ANOVA) was then used to test for differences in phases of play (6) by position (3) and then by quarter (4). When significant main effects were observed, pairwise comparisons with Tukey’s Honest Significant Difference test were applied. Effect sizes (Cohens *d*), calculated from the ratio of the mean difference to the pooled standard deviation, were also derived to provide a description of the practical differences between the position and quarter with respect to the phases of match-play. Values of <0.20, 0.21–0.60, 0.61–1.20, 1.21–2.00 and >2.01 represented trivial, small, moderate, large and very large differences, respectively [29]. Statistical analysis was performed using R statistical software (R.3.2.2, R Foundation for Statistical Computing) or Microsoft Excel. Significance was set at *p* ≤ 0.05 for the linear models. Data is presented as mean ± 95% confidence intervals (CI) for all physical performance data.

## 3. Results

**Figure 1:**
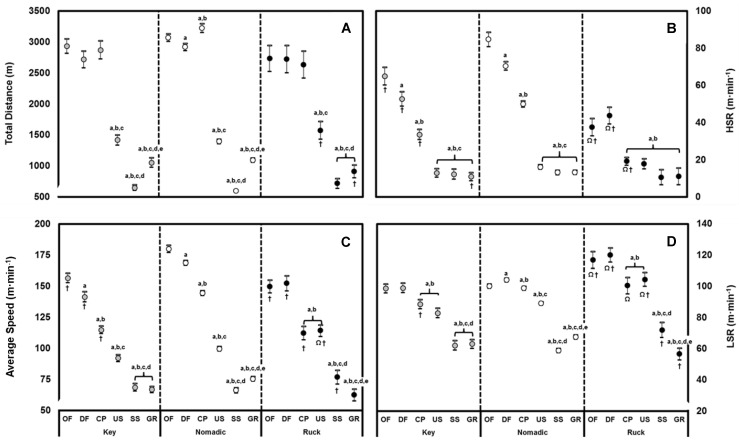
Positional analysis of (**A**) total distance (m), (**B**) average speed (m·min^−1^), (**C**) high-speed running (m·min^−1^) and (**D**) low-speed running (m·min^−1^) during the phases of professional Australian Football match-play. Values are mean ± 95% CI. Key: OF = Offence, DF = Defence, CP = Contested Play, US = Umpire Stoppage, SS = Set Shot, GR = Goal Reset. ^a^ Significantly different to OF, ^b^ Significantly different to DF, ^c^ Significantly different to CP, ^d^ Significantly different to US, ^e^ Significantly different to SS. Ω = Significantly different to key position players, † = significantly different to nomadic players (*p* < 0.05).

Linear mixed models revealed significant main effects for all positions and quarters of match-play (F = 63.35, *p* < 0.001). Figure 1 shows that for key position players, no differences existed between offence, defence, and contested play for TD (*d*: 0.09–0.33). Collectively, these phases of match-play comprised 73% of TD covered per match. Umpire stoppages consisted of 12% of TD, while goal resets and set shots comprised of 9% and 6%, respectively. Average speed was higher in offence compared to defence, contested play, and umpire stoppages (*d*: 0.55, 1.81, 2.96). No differences existed between set shots and goal resets (*d* = 0.11). HSR followed a similar profile (*d*: 0.51–1.71), although no difference existed between umpire stoppage, set shot, and goal reset (*d*: 0.12–0.32). Both offence and defence demonstrated higher LSR than the remaining phases of play (*d*: 0.79–3.12). No differences in LSR were exhibited when comparing contested play with umpire stoppages (*d* = 0.37), set shot (*d* = 0.08), and goal reset (*d* = 0.08).

For nomadic players, small to very large differences were observed between phases with respect to TD (*d*: 0.20–4.93). TD was highest in contested play (26%), followed by offence (25%), defence (24%), umpire stoppage (11%), goal reset (9%), and set shot (5%). All phases of play differed for average speed (*d*: 0.22–5.06) with the highest being offence, followed by defence and contested play. HSR followed a similar trend with higher values exhibited in offence compared to defence (*d* = 0.26) and contested play (*d* = 1.38). HSR during contested play was higher than umpire stoppages (*d* = 2.43), while no difference existed between set shot and goal reset (*d* = 0.12). Low-speed defensive running was greater than offence and contested play (*d*: 0.24–0.37). Umpire stoppages involved more LSR compared to goal reset and set shot (*d*: 1.55–2.16).

**Figure 2:**
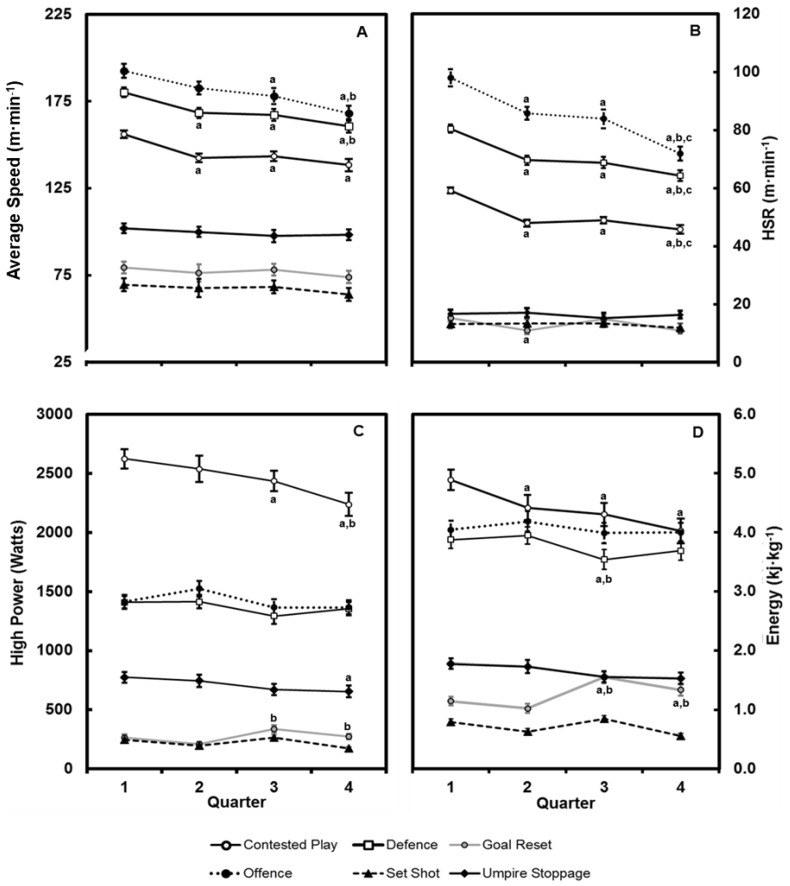
Match analysis by quarter for (**A**) average speed (m·min^−1^), (**B**) high-speed running (m·min^−1^), (**C**) high power (watts) and (**D**) energy expenditure (kj·kg^−1^) during the phases of professional Australian Football match-play. Values are mean ± 95% CI. ^a^ significantly different to quarter 1, ^b^ significantly different to quarter 2, ^c^ significantly different to quarter 3.

For ruckmen, no differences were evident for TD between offence, defence and contested play (all 23–24%; *d*: 0.02–0.14) (Figure 1). However, umpire stoppages involved a higher proportion of TD when compared to goal reset and set shot (*d*: 1.66–2.37). Average speed did not differ between offence and defence (*d* = 0.12), however both demonstrated higher values than umpire stoppages and contested play (*d*: 0.64–1.29). Comparatively, no differences existed between phases for HSR. No differences were evident between offence and defence for LSR (*d* = 0.25) nor for contested play with umpire stoppages (*d* = 0.14). However, both contested play and umpire stoppage were higher than set shots and goal resets (*d* = 1.48, 2.69). Figure 2 shows temporal changes in physical performance measures by quarter and phase of play. Post hoc analysis revealed no differences in total field time for all quarters of match-play (*p* = 0.46, *d*: 0.12–0.20). However, total time in offence was higher in the second (*d =* 0.36) and fourth quarter (*d =* 0.64) when compared to the first. Additionally, the total time spent in contested play and umpire stoppages during the fourth quarter was less than the first (*d =* 0.23; 0.37). Compared to the first quarter, average speed decreased in the second, third and fourth quarter for offence (*d:* 0.51–0.90), defence (*d*: 0.63–0.89), and contested play (*d*: 0.77–0.92). There were no differences in average speed for umpire stoppages, goal reset and set shot among the four quarters (*d*: 0.22–0.25). Compared to the first quarter, reductions in HSR were evident in offence, defence, and contested play in the second (*d*: 0.35–0.78), third (*d*: 0.44–0.68) and fourth quarters (*d*: 0.72–0.78). Offensive running during the fourth quarter was less than all other quarters (*d*: 0.30–0.76). A reduction in HSR during goal resets was evident during the second (*d =* 0.32) and fourth quarters (*d =* 0.56) only, while no differences were observed for umpire stoppages and set shots. There was a reduction in HP during the fourth quarter (*d =* 0.66) for contested play, while increases were observed during the third (*d =* 0.75) and fourth quarter (*d =* 0.35) during goal resets. In contrast, there were no differences in HP observed for offence, defence, umpire stoppages, and set shots (*d*: 0.03–0.19). Energy expenditure during the first quarter was higher than all other quarters during contested play (*d*: 0.37–0.70), while no differences were observed during offence. A reduction in energy expenditure was observed in the third quarter compared to the first (*d =* 0.33) and second quarter (*d =* 0.40) during defence. In contrast, an increase was observed in the third (*d*: 0.71–0.91) and fourth quarter (*d*: 0.34–0.55) for goal resets compared to the opening two quarters.

## 4. Discussion

This study examined the influence of the phases of AF match-play when considering player position and game quarter in a professional team via the use of integrated video and GPS analysis. The results revealed that speed and distance variables differed both within and between player positions. Reductions in speed-based and metabolic power-based estimates during offence, defence, and contested match-play were evident, however, this was not the case during stoppage periods. Collectively, these results suggest that running performance during the phases of match-play are influenced by player position and game quarter during competitive matches. There are several practical outcomes arising from these findings that have implications for sport science and conditioning staff.

It is widely accepted that nomadic positions in AF involve higher running demands compared to other positions [2,3,17]. Equally, the current results demonstrated that nomadic players had the highest total distance and average speeds during offence, defence, and contested match-play when compared to other positions. Furthermore, LSR and HSR were highest during these phases of match-play which is congruent with other lines of enquiry [19]. While limited information exists regarding positional movement profiles during the phases of match-play, these results are not surprising given nomadic players have lower body mass, higher aerobic fitness ability and are interchanged more frequently [2,30]. Additionally, nomadic players typically have a direct influence on the team’s offensive and defensive running patterns, indicating high levels of involvement with determining overall match-play intensities. Thus, it appears pertinent to consider the timing of rotations for teams, especially for nomadic players, to maintain HSR performance during the offensive and defensive elements of AF match-play.

In the present study, key position players had similar movement profiles to nomadic players during the phases of match-play; however, average speed and HSR intensities were lower. These results concur with previous research where tall backs and tall forwards elicited less overall running than all other positions except for ruckmen [22]. Indeed, key position players typically remain in their defensive or offensive positions on the field to maintain team structures and thus, these movement demands possibly reflect a lesser involvement in active periods of match-play due to their fixed positioning on the field. Nevertheless, key position players often change positions during the match depending on the team’s need to increase offensive or defensive numbers. This evidence suggests that the movement profiles of key and nomadic position players are similar, however the intensities performed in each phase of match-play differ. Key position and nomadic players could share similar training approaches, however, considerations to total workloads, intensities and technical skill demands are recommended.

The results of this study highlight the specific movement profiles of ruckmen during professional AF competition [4], as demonstrated by the different profiles between each phase of match-play. Interestingly, ruckmen performed more LSR during offence, defence and umpire stoppages than nomadic and key position players. Additionally, higher average speeds were observed for ruckmen during umpire stoppages. Ruckmen typically attend each stoppage anywhere on the field to compete with the opposition ruckmen and assist the nomadic players to win the ball during contested match-play. Indeed, Dawson et al. [15] reported that ruckmen, along with midfield players, have the highest frequency of involvement in game activities, i.e., possessions, ruck duels, ground ball contests, shepherds, spoils, bumps and tackles during the game. The difference in running demands for the various phases of play were smaller for ruckmen when compared to the within playing group comparisons of nomadic and key position players. These findings provide evidence of a more continuous running profile for ruckmen when considering the phases of match-play which has implications for training and conditioning approaches to this specific position in AF.

Previous AF studies have reported reductions in movement profiles across the four quarters of match-play [2,3,21]. Similarly, our results show decrements in running intensities for both speed-based and metabolic power estimates over the match. However, the reductions in running performance were not identical among the various phases of match-play. Generally, our results revealed that HSR was reduced during offence, defence and contested match-play in the second, third and fourth quarter compared to the first. For the most part, this suggests that running performance is maintained during stoppages across the four quarters, while reductions in movement intensities are isolated to periods of active match-play. These results differ from previous findings where AF players reduced low-intensity activity to potentially maintain high-intensity movements during the later periods of match-play [3,31]. It is possible that the evolution of training practices, tactics adopted by either team or relative caliber of the investigated teams may explain this discrepancy, nonetheless, there are notable implications for training prescription originating from these findings.

The present results also show that reductions in speed and distance were only synonymous with metabolic power indices during contested play and not during the other analysed phases. HP and energy expenditure generally did not change over the four quarters during offence and defence phases. Except for contested play, these findings suggest that accelerated and decelerated running appears to be maintained over the four quarters during each phase of match-play. In fact, HP and energy expenditure increased during goal resets in the third and fourth quarter when compared to the first and second. These results may be due to positional changes during the later stages of the match due to injury, increased rotations, or changes to team structures. Furthermore, recent research has highlighted the value of metabolic power indices in capturing accelerated power efforts during scenarios where spatial constraints may limit player opportunity to reach high speed thresholds [2]. Indeed, the distance and speed profiles differed from the metabolic power profiles over the four quarters. Specifically, HP and energy expenditure were notably higher during contested play where players compete for possession of the ball in congested scenarios. Notwithstanding the reliability considerations of these metrics [32], these findings may have implications for elements of competition or training that involve a high proportion of contested play, for example, during wet weather games, where HSR performance may be limited. Clearly, the incorporation of metabolic power estimates can provide valuable information about AF match-play.

This study examined the influence of player position and match quarter during the various phases of match-play in AF with wide-ranging applications from the findings. Nevertheless, there are limitations and further research questions related to this study that should be addressed. A single team participated in this study and thus, the results may not be generalisable to other teams competing in the AFL or lower tiers of competition. Nevertheless, the data utilised in this research was from a high caliber team that competed in the final’s series, thus representing a high level of data ecological validity. Future studies involving multiple teams would provide a greater basis for determining whether differences exist between successful and unsuccessful teams. Additionally, several factors may influence the movement profiles during the various phases of match-play including environmental conditions, caliber of the opposition, match location and within match point differential that may all contribute to the changes in quarter-by-quarter running profiles Future studies should assess the interaction effects of these factors to further understand the demands of the various phases of match-play. The prevalence of new technology is commonplace in professional sport. New devices that measure more discrete movement such as integrated inertial movement devices could offer more insight into changes in locomotion and fatigue. A number of recent studies have highlighted the value of inertial movement sensors in the measurement of coordinative movement including kicking in soccer [33], running mechanics [34] and tennis strokes [35]. These studies exhibit the potential for more specific detail to be elucidated within team sport competition. One of the limitations of this study was the indirect measurement of fatigue, which was inferred from the speed, distance and metabolic power indices. Along with measures of internal load, inertial measurements may provide more insight and understanding into transient fatigue in professional AF.

## 5. Conclusions

This study assessed the influence of position and quarter on the match running demands during the phases of AF match-play in a professional team. Speed and distance physical performance indices differed within positions and the highest intensities performed by all positions were generally during offence, defence and then contested match-play. The nomadic players had the highest game intensities, while ruckmen differed to nomadic and key position players. Additionally, reductions in speed and metabolic power metrics were evident across the four quarters of match-play during offence, defence and contested play, but not during stoppage periods. Collectively, this study demonstrates that physical performance during the phases of match-play are influenced by player position and game quarter during competitive matches.

## Data Availability

The data is not publicly available due to restrictions from the club where the data was obtained and will be shared upon specific request.
